# The Nepalese health care system and challenges during COVID-19

**DOI:** 10.7189/jogh.11.03030

**Published:** 2021-02-11

**Authors:** Prajwal Neupane, Dilip Bhandari, Masaharu Tsubokura, Yuzo Shimazu, Tianchen Zhao, Koji Kono

**Affiliations:** 1 Department of Gastrointestinal Tract Surgery, Fukushima Medical University School of Medicine, Fukushima City, Fukushima, Japan; 2 Department of Neurosurgery, Fukushima Medical University School of Medicine, Fukushima City, Fukushima, Japan; 3 Department of Radiation Health Management, Fukushima Medical University School of Medicine, Fukushima City, Fukushima, Japan

Nepal faces a massive burden from coronavirus disease 2019 (COVID-19). The first case of COVID-19 was identified in Wuhan, China, on 31 December 2019, whereas the first case of COVID-19 in Nepal was reported on 24 January 2020 [1]. Since the inception of the virus, the number of cases has continued to increase and the health care system in Nepal is greatly affected by COVID-19. As of 3 January 2021, there were 261 859 total recorded cases, 254 494 recovered cases, 1878 deaths, and 5487 active cases [2]. The major challenges faced while providing quality health care during the COVID-19 pandemic are discussed below.

## HEALTH CARE WORKERS AND HEALTH CARE INSTITUTES

An enormous number of health care workers is needed to combat the COVID-19 pandemic. However, with a population of 28.087 million, Nepal was already facing a shortage of medical doctors, nurses, and paramedics before the inception of COVID-19 [3,4]. As of September 2016, the number of total health care personnel including doctors, nurses, and midwives in Nepal was 3.15 per 1000 population [5]. As per government data, the numbers of intensive care beds and advanced respiratory support (ventilators) are limited to 1395 and 480 respectively [6]. These ICU beds and ventilators are for both COVID and non-COVID patients. An article published in *The New York Times* also emphasised the shortage of ICU beds and ventilators and stated that some patients had died in ambulances while searching for ICU beds [7].

## LOCKDOWN AND SOCIOECONOMIC CONDITIONS

The percentage of the Nepali population living below the national poverty line was 25.2% in 2010 [8]. COVID-19-related disruptions in livelihoods and the contraction in household consumption are expected to disproportionately affect the poor, vulnerable, and households engaged in informal sector activities, and poverty is likely to increase in 2020 [9]. A series of lockdowns and travel restrictions was imposed from 24 March to 21 July 2020 in order to contain the spread of the virus at the community level. Working class people who were paid wages on a daily basis were greatly affected by this situation. Living with extreme poverty and without food, they were forced to eat food provided by INGOs, NGOs, and other volunteers on the streets and at public gatherings, which made them more vulnerable to contact and spreading COVID-19. The health ministry has declared free health services for such people who cannot afford the price of treatment for COVID-19.

## OTHER HEALTH PROBLEMS

Providing quality health care during the COVID-19 pandemic has been challenging and such care is limited to only a few people. Nepal faces more challenges in providing health care services than developed countries. In the context of Nepal, factors such as poverty, illiteracy, lack of infrastructure, shortage of health care professionals, attitude towards medical professionals, security concerns of health care professionals, health insurance policies, geographic distribution, culture, governmental policies, and physical barriers also directly affect health care services being provided [10,11]. Health indicators like the infant mortality rate (IMR), maternal mortality rate (MMR), under-five mortality rate (U5MR), and average life expectancy have improved when compared to 2010; the IMR and U5MR were 37.79 and 46.98 per thousand live births in 2010 and 26.36 and 31.55 per thousand in 2019, respectively. The MMR was 305 per 100 000 in 2010, which improved to 186 per 100 000 in 2017 [12]. However, these parameters again seem to be worsening during the COVID-19 pandemic; while existing programmes such as the Safe Motherhood programme were affected by COVID-19, in a study published In *The Lancet Global Health,* Ashish and colleagues reported that “Neonatal deaths increased from 13 deaths per 1,000 livebirths before lockdown and reached 40 deaths per 1,000 livebirths during lockdown, and institutional stillbirths increased from 14 per 1,000 total births before lockdown to 21 per 1,000 total births during lockdown”[13]. The major health problems that account for the majority of deaths include non-communicable diseases (eg, intracerebral haemorrhage, COPD, and ischemic heart diseases) [14]. Addressing these health problems during this COVID-19 pandemic is also necessary and programmes to cover other diseases should be initiated as soon as possible.

## WHAT CAN BE DONE?

During this COVID-19 pandemic, decentralising health care facilities in remote districts; establishing new, well-equipped, advanced, specialised health care institutions across the country; uplifting the current conditions of governmental hospitals and providing better health services; providing free or minimal charges for health services (whichever is feasible); developing medical education systems and qualified health professionals; providing security, proper salaries, and benefits for health care workers; and implementing government policies to ensure basic needs for poor and affected individuals, are required. However, implementing all of the above suggestions within Nepal’s present economy will be difficult. Foreign grants and aid with technical support can be helpful.

**Figure:**
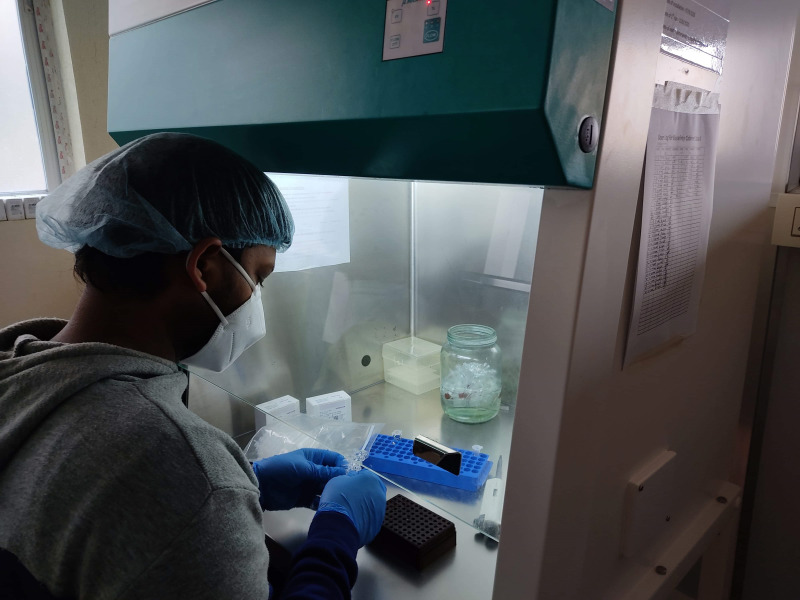
Photo: from the collection of Dr. Pratik Neupane (used with permission).

There are lessons to be learned from countries like Italy, which was severely affected by the current COVID-19 pandemic. Governments can act appropriately if they adopt certain suggestions: First, health care systems’ capacity and financing need to be more flexible to take into account exceptional emergencies. Second, in response to emergencies, solid partnerships between private and public sectors and the government should be institutionalised. Third, recruitment of health care workers and other human resources must be planned and financed according to a long-term vision. Fourth, decentralisation of health care institutions throughout the country and mutual coordination between health ministry and its divisions should be established. Consistent management choices and a strong political commitment are needed to create a more sustainable system [15].

The best health services can be received and given only when the relationships between patient, doctors, health professionals, health care institute and government are run with mutual coordination and understanding.
